# Supplementary material for: MetaRibo-Seq measures translation in microbiomes
Source: Nat Commun. 2020 Jun 29;11:3268. doi: 10.1038/s41467-020-17081-z (PMC7324362; doi:10.1038/s41467-020-17081-z)
Supplement: Supplementary file 10 — Supplementary Data 7 [file 41467_2020_17081_MOESM10_ESM.zip › File2/Confidence_VeryHigh_Taxonomy/85549_out.krona.html]

Javascript must be enabled to view this page.

members
magnitude
magnitudeUnassigned
count
unassigned
taxon
rank

85549\_out

6

superkingdom
6
2

6
phylum
1239

186801
6
class

186802
order
6

31979
family
5

genus
5
1485


SRS077552\_contig\_number\_14297
1262824
1
species

1262805

SRS014287\_contig\_number\_8751
1
species

2292993

SRS019068\_contig\_number\_78986SRS148159\_contig\_number\_41333
2
species

species
1
2293051

SRS049712\_contig\_number\_18898

186803
family
1

1506553
1
genus


SRS146888\_contig\_number\_contig-100\_639.93381
1512
species
1
